# CD271 Defines a Stem Cell-Like Population in Hypopharyngeal Cancer

**DOI:** 10.1371/journal.pone.0062002

**Published:** 2013-04-23

**Authors:** Takayuki Imai, Keiichi Tamai, Sayuri Oizumi, Kyoko Oyama, Kazunori Yamaguchi, Ikuro Sato, Kennichi Satoh, Kazuto Matsuura, Shigeru Saijo, Kazuo Sugamura, Nobuyuki Tanaka

**Affiliations:** 1 Division of Cancer Biology and Therapeutics, Miyagi Cancer Center Research Institute, Natori, Miyagi, Japan; 2 Molecular and Cellular Oncology, Miyagi Cancer Center Research Institute, Natori, Miyagi, Japan; 3 Cancer Stem Cell, Miyagi Cancer Center Research Institute, Natori, Miyagi, Japan; 4 Department of Head and Neck Surgery, Miyagi Cancer Center, Natori, Miyagi, Japan; 5 Department of Pathology, Miyagi Cancer Center, Natori, Miyagi, Japan; 6 Cancer Pathology, Department of Cancer Science, Tohoku University Graduate School of Medicine, Sendai, Miyagi, Japan; Cincinnati Children's Hospital Medical Center, United States of America

## Abstract

Cancer stem cells contribute to the malignant phenotypes of a variety of cancers, but markers to identify human hypopharyngeal cancer (HPC) stem cells remain poorly understood. Here, we report that the CD271^+^ population sorted from xenotransplanted HPCs possesses an enhanced tumor-initiating capability in immunodeficient mice. Tumors generated from the CD271^+^ cells contained both CD271^+^ and CD271^−^ cells, indicating that the population could undergo differentiation. Immunohistological analyses of the tumors revealed that the CD271^+^ cells localized to a perivascular niche near CD34^+^ vasculature, to invasive fronts, and to the basal layer. In accordance with these characteristics, a stemness marker, *Nanog*, and *matrix metalloproteinases (MMPs)*, which are implicated in cancer invasion, were significantly up-regulated in the CD271^+^ compared to the CD271**^−^** cell population. Furthermore, using primary HPC specimens, we demonstrated that high CD271 expression was correlated with a poor prognosis for patients. Taken together, our findings indicate that CD271 is a novel marker for HPC stem-like cells and for HPC prognosis.

## Introduction

Head and neck squamous cell carcinoma (HNSCC) is the sixth most common cancer worldwide, with nearly 500,000 new cases and an estimated 300,000 deaths reported every year. This term includes various independent cancers, such as oral, nasopharyngeal, oropharyngeal, and hypopharyngeal cancers [1]. Hypopharyngeal cancer (HPC), a malignancy of the hypopharynx, accounts for approximately 10% of all HNSCCs. Epidemiological studies indicate that tobacco and alcohol consumption contribute to its carcinogenesis [2]. Unfortunately, about 80% of HPC cases are advanced at the time of diagnosis, i.e., the patients are in stage III or IV [3], so treatment is difficult. Radical surgery such as total laryngopharyngoesophagectomy results in loss of the voice, and concurrent chemoradiotherapy often causes life-threatening side effects. Delayed regional lymph node metastasis, distant metastasis, and additional primary malignancies frequently occur during the course of the disease [3]. The 5-year survival rate of HPC patients is no more than 30% [4], suggesting a strong need for innovative treatment strategies.

The accumulating evidence of recent years supports the cancer stem cell (or initiating cell) theory [5], [6]. In this fascinating scenario, cancers are composed of a hierarchy of heterogeneous cell populations, and are initiated from a limited subpopulation of cells with stem cell-like properties. With their self-renewal activity and tumor-initiating capability, cancer stem cells (CSCs) or cancer initiating cells (CICs) generate non-cancer stem cells as an ‘Offspring’ population. Resistance to chemotherapy and radiotherapy is another characteristic of CSCs. Thus, CSCs can be responsible for treatment failures of chemotherapy and radiotherapy, and poor clinical outcomes. Toward the goal of developing new CSC-targeting therapies, researchers have sought to identify and characterize cell-surface markers for CSCs.

In HNSCC, CD44^+^ cells have been identified as CSCs. When clinical cancer specimens are xenografted into immunodeficient mice, CD44^+^ cells but not CD44^−^ cells initiate tumors, and the resulting tumors generate a hierarchy of heterogeneous cell populations [7]. However, a recent study showed that CD44^−^ cells form spheres, possess tumor-initiating capability, and are chemoresistant, like CD44^+^ cells [8]. Thus, the CSCs may be dynamic and heterogeneous in various microenvironments [9], and the CD44^+^ cells may not represent pure HNSCC CSCs. In addition, HNSCC itself is heterogeneous, representing various cancer types, as described above. Therefore, targeting research for each cancer type may lead to the identification of additional CSC markers.

CD271 is a transmembrane protein that belongs to the tumor necrosis factor receptor superfamily. It is also a nerve growth factor receptor (NGFR), and interacts with neurotrophins, such as NGF, brain-derived neurotrophic factor (BDNF), neurotrophin-3 (NT3), and neurotrophin-4 (NT4), with low affinity [10]. Former studies suggest that the tissue stem cells of oral [11], laryngeal [12], and esophageal [13] squamous epithelia express CD271. CD271 is also associated with the CSCs of malignant melanoma [14], [15] and esophageal squamous cell carcinoma [16].

In the present study, we demonstrate that the CD271^+^ cell population of HPC possesses tumor-initiating capability *in vivo*, and has several CSC-like characteristics.

## Materials and Methods

### Tumor Implantation

After obtaining informed consent, fresh tumor specimens were obtained at the Miyagi Cancer Center (MCC), transported to the laboratory in cooled PBS(−), and subjected to further analyses. All tumor samples were anonymized in accordance with the MCC Institutional Review Board. NOD/SCID/IL-2RγC^null^ (NOG) mice purchased from the Central Institute for Experimental Animals Japan were anesthetized with pentobarbital. Once the mice were asleep, skin incisions were made, and small pieces of tumor specimens about 50 mm^3^ were implanted under the skin of the flank on both sides. Tumor formation was monitored weekly, and the tumor volume was calculated by the formula: 1/2×vertical range×horizontal range×height. When the original tumors in the xenotransplanted NOG mice reached over 10 mm in diameter, the mice were sacrificed, and the tumors were divided into samples for single-cell digestion, sphere formation, formalin fixation for histology, or serial passage in mice. The animal care and experimental protocols were performed in strict accordance with the procedures and guidelines established by the MCC administrative panels for laboratory animal care. All surgeries were performed under anesthesia, and all efforts were made to minimize suffering.

### Preparation of Single-cell Suspensions from Tumor Tissue Specimens

Under aseptic conditions, tumors were cut into small fragments, and finely minced with a sterile scalpel. After being washed with PBS(−), the tumor tissue was soaked in a solution containing PBS(−), 1 mg/ml DNase1 (Roche), and 1 mg/ml Collagenase/Dispase (Roche), and incubated at 37°C for 2 or 3 hours, until complete digestion had occurred. The cells were passed through a 40-µm nylon mesh, and washed 3 times with PBS(−). After centrifugation, the pool of single cells was divided, and the cells were used for FACS analyses or sphere culture. For sphere culture, the cells were suspended in serum-free DMEM/F12 supplemented with B27 (Invitrogen), human recombinant epidermal growth factor (EGF: 20 ng/µl: Peprotech), and human basic fibroblast growth factor (bFGF: 20 ng/µl: Peprotech) on ultra-low attachment culture dishes (Corning).

### Flow Cytometry Analysis and Cell Sorting

Dissociated single cells were suspended in PBS(-) with 3% FBS. The cells were stained with anti-human specific EpCAM antibodies (1∶10, FITC-conjugated, Miltenyi Biotec), anti-human CD271 antibodies (1∶10, APC-conjugated, Miltenyi Biotec), anti-human CD44 antibodies (1∶10, APC-conjugated, Becton Dickinson), anti-human CD133 antibodies (1∶10, APC-conjugated, Miltenyi Biotec), and anti-mouse IgG1, κ isotype control antibodies (1∶20, APC and FITC-conjugated, Biolegend and Becton Dickinson, respectively). 7-AAD was used to exclude dead cells (Sigma, 1 µg/ml). The stained cells were either analyzed with a FACSCanto™ II (Becton Dickinson) or sorted with a FACSAria™ II (Becton Dickinson), in accordance with the manufacturer’s protocol.

### Real-time Reverse Transcription (RT)-PCR

The total RNA was purified from sorted single cells or tissue specimens using a mirVana™ miRNA Isolation Kit (Ambion), and transcribed using a PrimScript II cDNA Synthesis Kit (Takara Bio). Real-time RT-PCR was performed using Brilliant III Ultra-Fast SYBR Green QPCR Master Mix (Agilent Technologies). *GAPDH* was used as an endogenous reference gene. The primer sequences used for real-time RT-PCR are listed in **Table S1**.

### Immunohistochemistry (IHC)

Paraffin-embedded, formalin-fixed, 3-µm tissue sections were deparaffinized in xylene, and rehydrated through ethanol to distilled water. Heat-induced epitope retrieval was performed by microwaving sections in a pH 9.0 target retrieval solution (Dako). The endogenous peroxidase was blocked with 0.3% H_2_O_2_. The sections were incubated with primary antibodies to human CD271 (1∶4000, BD Biosciences) for 20 min, or to CD34 (Nichirei Biosciences) or Ki-67 (1∶10, Santa Cruz Biotechnology) for 60 min, at 37°C. The sections stained for CD271 were incubated for 15 min with mouse LINKER (Dako), then secondary antibodies and DAB Chromogen (Envision™ FLEX Kit, Dako) were applied as described in the manufacturer’s protocol. To the sections stained for CD34 or Ki-67, Simple Stain AP (M) (Nichirei Biosciences) was applied as the secondary antibody, and the staining was visualized with New Fuchsin Substrate (Nichirei Biosciences). For the double staining of CD34 or Ki-67,with CD271, the CD34 or Ki-67 staining was performed first, followed by that for CD271, as described above.

### *In vivo* Tumorigenesis Assay

Dissociated tumors were sorted based on the human EpCAM and CD271 expression, as EpCAM^+^ CD271^+^ cells or EpCAM^+^ CD271^−^ cells. The sorted cells were suspended in 200 µl of Matrigel matrix (BD Biosciences) at 4°C, then subcutaneously injected into the flanks of NOG mice with a 1-ml syringe. Each mouse received CD271^+^ cells in the right side, and CD271^−^ cells in the left. Tumor formation was monitored by weekly inspection and palpation.

### *In vivo* Chemotherapy Assay

Cisplatin (CDDP), an anti-cancer drug classified as a platinum reagent, was administered intravenously or intraperitoneally at 5 or 7.5 mg/kg. One week later, the mice were euthanized, and the tumors were extracted. The tumors were divided and either fixed with formalin for IHC, or dissociated into single cells and subjected to FACS analysis.

### Statistics

The analyses of disease-specific survival and relapse-free survival were conducted with Kaplan-Meier methods, and the log rank test was used to evaluate the difference between groups. Fisher’s exact test was used to compare two groups (“strong” versus “moderate-to-weak” CD271 expression) in resected tumors from 28 cases of HPC, and the chi-square test was used to compare the same two groups in the IHC study of 83 HPC cases. The average values of *Nanog* expression between the two groups was analyzed with Student’s t-test. The level of significance was set at *p*<0.05.

### Ethics

The Institutional Review Board of the MCC approved this study protocol, and written informed consent was obtained from each subject. The protocol of animal experiments was approved by the MCC Animal Care and Use Committee (Permit Number: MCC-AE-2011-8).

## Results

### HPC Tumors Include a Subpopulation of CD271^+^ Cells

To investigate tumor initiation and cancer stem cells, fresh primary tumor specimens obtained from HPC patients undergoing surgery were implanted under the skin of 8-10-week-old NOG mice. Three independent human HPC specimens with similar clinical characteristics were used to establish primary xenograft lines (HPCM1-3) (**Table S2**). The histology of each xenograft was consistent with its primary sample (**Figure S1**).

**Figure 1:**
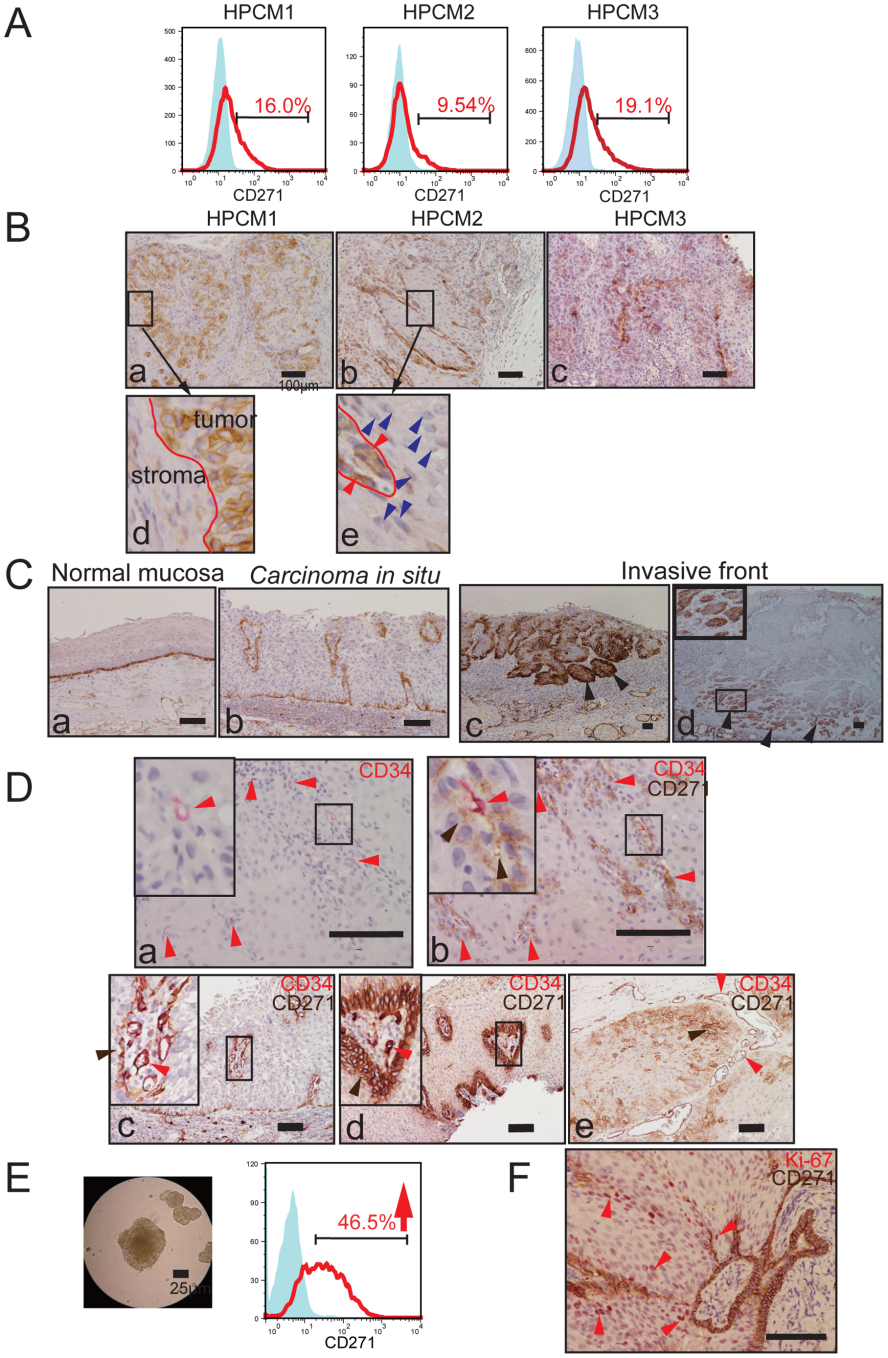
Distribution of CD271^+^ cells in HPC. (A) Cells derived from three HPC xenotransplanted lines were stained for CD271 and analyzed by FACS. (B) Tumor tissues dissected from mice transplanted with the three HPC lines were analyzed for CD271 expression by IHC. Immunopositivity appears brown (a, b, and c). High-magnification images are linked to their respective boxed areas by arrows. Scale bar: 100 µm. Red line indicates the border of the tumor and stroma (d). Red line indicates the border of a CD271^+^ cell cluster and CD271^−^ cells, and red and blue arrowheads show CD271^+^ cells and CD271^−^ cells, respectively (e). (C) Representative results of IHC for CD271 in clinical specimens. Normal mucosa (a), carcinoma *in situ*. (b). Arrowheads indicate an invasive front, and strongly positive CD271 expression (c, d). (D) IHC for CD34 with New Fuchsin substrate (a), and double staining for CD34 (New Fuchsin) and CD271 (DAB) (b-e). CD34 immunopositivity appears red. Insets show high-magnification images of the boxed areas. Red arrowheads indicate CD34-positive microvessels, and brown arrowheads indicate CD271^+^ cells. (E) Sphere-forming cells of HPCM1, and elevated CD271 expression in the FACS analysis. Scale bar: 25 µm. (F) IHC for the double staining of Ki-67 (New Fuchsin) and CD271 (DAB). Ki-67 immunopositivity appears red in the nucleus. Red arrowheads indicate Ki-67-positive cells. Scale bar: 100 µm.

To characterize the serially xenotransplanted tumor lines, their cell-surface marker expression was analyzed. Each tumor was extracted from the host and prepared as a single-cell suspension. Because EpCAM is reported to be a diagnostic tumor-cell marker for HNSCC [17], the human EpCAM-positive cells were strictly gated to eliminate host-derived cells, and analyzed by flow cytometry. Among the CSC markers tested, the cells were negative for CD133, and 3.4% of the tumor cells were positive for CD44 (data not shown). Unexpectedly, we observed a CD271^+^ subpopulation among the three HPC lines tested (**Figure 1A**). In repeated experiments, the CD271^+^ population represented 2.99 to 20.1% of the cells in HPCM1. Similar CD271^+^ populations were clearly present in the other two lines: 2.90–9.54% for HPCM2 and 19.1% for HPCM3. Next, we analyzed paraffin-embedded sections of tumors derived from the xenotransplanted HPC lines for CD271 by IHC (**Figure 1B**). Within the typical squamous cell carcinomas formed by the three lines, CD271^+^ cells were mainly present in the basal layer of the tumor, almost restricted to the peripheral zone of the tumor nest, and scarce in the center portion (**Figure 1B**). Under high magnification, CD271^+^ cells were observed next to the stroma (**Figure 1B** **–d**). These CD271^+^ cells exhibited morphologically immature phenotypes, whereas the CD271^−^ cells in the central zone of the tumor nest had more mature, flattened, and keratinized shapes (**Figure 1B** **–e**). To further characterize the localization of CD271^+^ cells within the HPC, we stained cytokeratins (CKs) in serial sections (**Figure S2**). CK5/6 tends to localize to basal layers and proliferating suprabasal compartments, and the CD271^+^ cells were localized within the CK5/6-positive region. The CD271^+^ cells were also moderately positive for CK8, which marks undifferentiated SCC cells. These results suggest that the CD271^+^ population is included in the basal-layer portion of the CK5/6-positive area, and partially overlaps with CK8-positive undifferentiated cells.

We also examined the CD271 expression within clinically dissected HPC specimens. First, we confirmed that CD271^+^ cells were present in the most basal layer of normal hypopharyngeal mucosa (**Figure 1C** **–a**). Similarly, in a carcinoma *in situ* (*CIS*) specimen, CD271^+^ cells were restricted to the basal layer, and were absent from the more differentiated upper layers (**Figure 1C** **–b**). Typically, most of the cancer cells located in the invasive front were CD271^+^ (**Figure 1C** **–c,d**). We also examined whether CD271^+^ cells were located close to the vasculature within tumors. We found that CD34^+^ microvascular endothelial cells (red in **Figure 1D**) were located in the CD271^+^ cell areas, and magnified images showed that some CD34^+^ and CD271^+^ cells were near each other, or in direct contact (**Figure 1D** **–a,b**). Of note, in most of the specimens, the CD271^+^ cells formed dense clusters that were surrounded by stroma, which contained CD34^+^ microvessels (**Figure 1D** **–c,d,e**). Together, these data indicated that CD271^+^ cells are present in the invasive front and in the perivascular area of tumors.It is well known that cells from various cancers, including HNSCC, that form spheres under suspension culture conditions include CSCs [18]. Hence, we examined whether sphere-formation culture would affect the CD271 expression. Dissociated cancer cells generated spheres that survived for 12 days, and single cells prepared from these spheres were subjected to CD271 analysis (**Figure 1E**). CD271 was expressed in 46.5% of these cells, in contrast to the original HPCM1 cell tumor, in which 2.99–20.1% of the cells were CD271^+^. This result suggested that *in vitro* sphere formation caused the enrichment of CD271^+^ cells.

We also examined whether the CD271^+^ cells are a proliferating population. Double -staining experiments with Ki-67 and CD271 showed that the CD271^+^ cells mostly resided in the basal layer, whereas the Ki-67-positive cells localized mainly to the relatively differentiated suprabasal layers (Figure 1F). These results suggest that the CD271^+^ cells are not actively proliferating *in vivo*.

### CD271^+^ Cells are Tumorigenic and Differentiate *in vivo*

**Figure 2:**
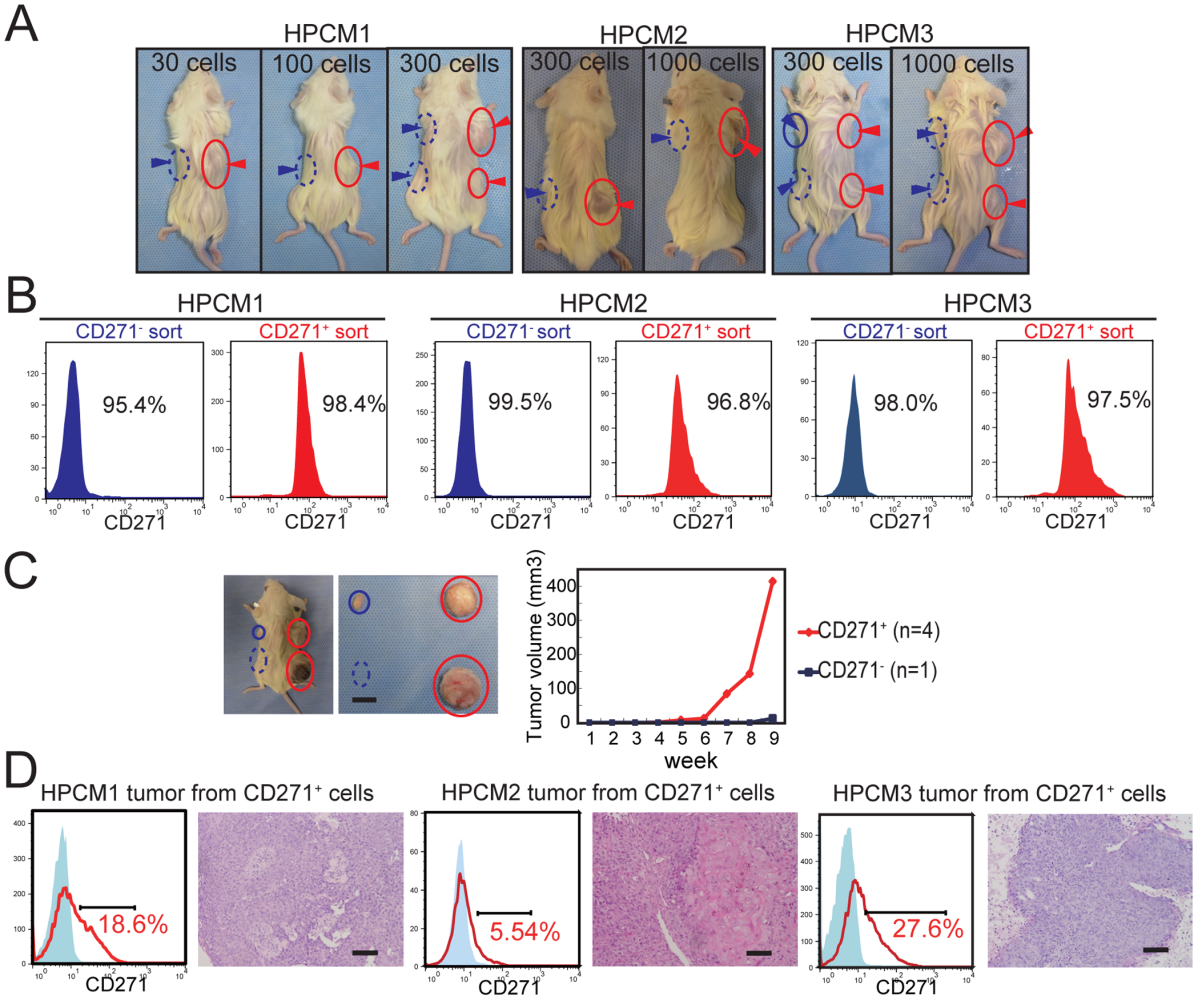
In vivo tumorigenicity and differentiation capacity of CD271^+^ cells. (A) Representative tumors in mice into which the indicated number of cells were transplanted. Red arrowheads indicate CD271^+^ cell injection sites (right side), and blue arrowheads indicate CD271^−^ cell injection sites (left side). Circles and dashed circles indicate transplantation locations resulting in success and failure of tumor formation, respectively. (B) Flow cytometry analysis of the sorted cells (96.8∼99.5% CD271^+^ or CD271^−^ cells). (C) Thirty CD271^+^ cells (right side) and CD271^−^ cells (left side) were transplanted into a mouse, and the generated tumors were resected. Tumor growth was plotted using the average value. (D) Flow cytometry analysis of the CD271 expression of tumor cells resulting from the injection of CD271^+^ cells. Tumor sections were stained with hematoxylin and eosin (H&E). Scale bar: 100 µm.

We next examined the *in vivo* tumorigenicity of the CD271^+^ cells of the three HPC lines. Thirty to 100,000 CD271^+^ or CD271^−^ cells were subcutaneously injected into each side of the same mouse to avoid any host/environmental differences (**Figure 2A**). The accuracy of sorting was confirmed by FACS analysis (**Figure 2B**). The xenotransplantation results are summarized in **Table 1**. In HPCM1, the CD271^+^ cells initiated tumors at an extremely high rate, even when fewer than 300 cells (but at least 30 cells) were injected. Thirty CD271^−^ cells generated a tumor in only one of six inoculations, and the tumor that formed was much smaller than those initiated by the CD271^+^ cells (**Figure 2C**). Similarly, for HPCM2, all the tumors that formed, except for one, arose from CD271^+^ cells. Although the CD271^−^ cells in HPCM3 generated tumors, they showed a longer latency and lower frequency than those that developed from CD271^+^ cells. These data indicated that the CD271^+^ cells possessed higher tumorigenicity than the CD271^−^ cells *in vivo*.

**Table 1:** Tumor initiation capability of CD271^+^ cells vs, CD271^−^ cells.

	Population	Number of cellsinjected	Weeks				
			2	4	6	8	10
HPCM1	CD271^+^	30	0/6	**1/6**	**2/6**	**4/6**	**4/6**
	CD271^−^	30	0/6	0/6	0/6	0/6	**1/6**
	CD271^+^	100	0/4	**1/4**	**3/4**	**3/4**	**3/4**
	CD271^−^	100	0/4	0/4	0/4	0/4	0/4
	CD271^+^	300	0/4	**1/4**	**4/4**	**4/4**	**4/4**
	CD271^−^	300	0/4	0/4	0/4	0/4	0/4
	CD271^+^	1,000	0/2	**1/2**	**2/2**	**2/2**	**2/2**
	CD271^−^	1,000	0/2	0/2	**2/2**	**2/2**	**2/2**
HPCM2	CD271^+^	100	0/4	0/4	0/4	0/4	0/4
	CD271^−^	100	0/4	0/4	0/4	0/4	0/4
	CD271^+^	300	0/6	0/6	0/6	**1/6**	**1/6**
	CD271^−^	300	0/6	0/6	0/6	0/6	0/6
	CD271^+^	1,000	0/8	**1/8**	**2/8**	**4/8**	**4/8**
	CD271^−^	1,000	0/8	0/8	**1/8**	**1/8**	**1/8**
	CD271^+^	10,000	0/2	**1/2**	**1/2**	**1/2**	**1/2**
	CD271^−^	10,000	0/2	0/2	0/2	0/2	0/2
HPCM3	CD271^+^	300	0/4	0/4	**1/4**	**2/4**	**3/4**
	CD271^−^	300	0/4	0/4	**1/4**	**1/4**	**1/4**
	CD271^+^	1,000	0/4	0/4	**3/4**	**4/4**	**4/4**
	CD271^−^	1,000	0/4	0/4	0/4	**2/4**	**2/4**
	CD271^+^	10,000	0/4	0/4	**4/4**	**4/4**	**4/4**
	CD271^−^	10,000	0/4	0/4	0/4	**3/4**	**4/4**
	CD271^+^	100,000	0/2	**2/2**	**2/2**	**2/2**	**2/2**
	CD271^−^	100,000	0/2	0/2	**2/2**	**2/2**	**2/2**

Morphologically, the CD271^+^ cells formed tumors that resembled the original one histologically, with typical SCC features: basal cell-like morphology in the peripheral zone, and differentiated cells in the central portion of the tumor nest (**Figure 2D**). The generated tumors were also examined for CD271 expression by flow cytometry. The tumors arising from the CD271^+^ cells contained both CD271^+^ and CD271^−^ cells (**Figure 2D**). Thus, the CD271^+^ population has the potential to generate a hierarchy of CD271-expressing and non-expressing cells, as well as the ability to initiate cancer.

### Gene Expression Profile of CD271^+^ Cells Reveals Some Characteristics of Stemness and Invasiveness

**Figure 3:**
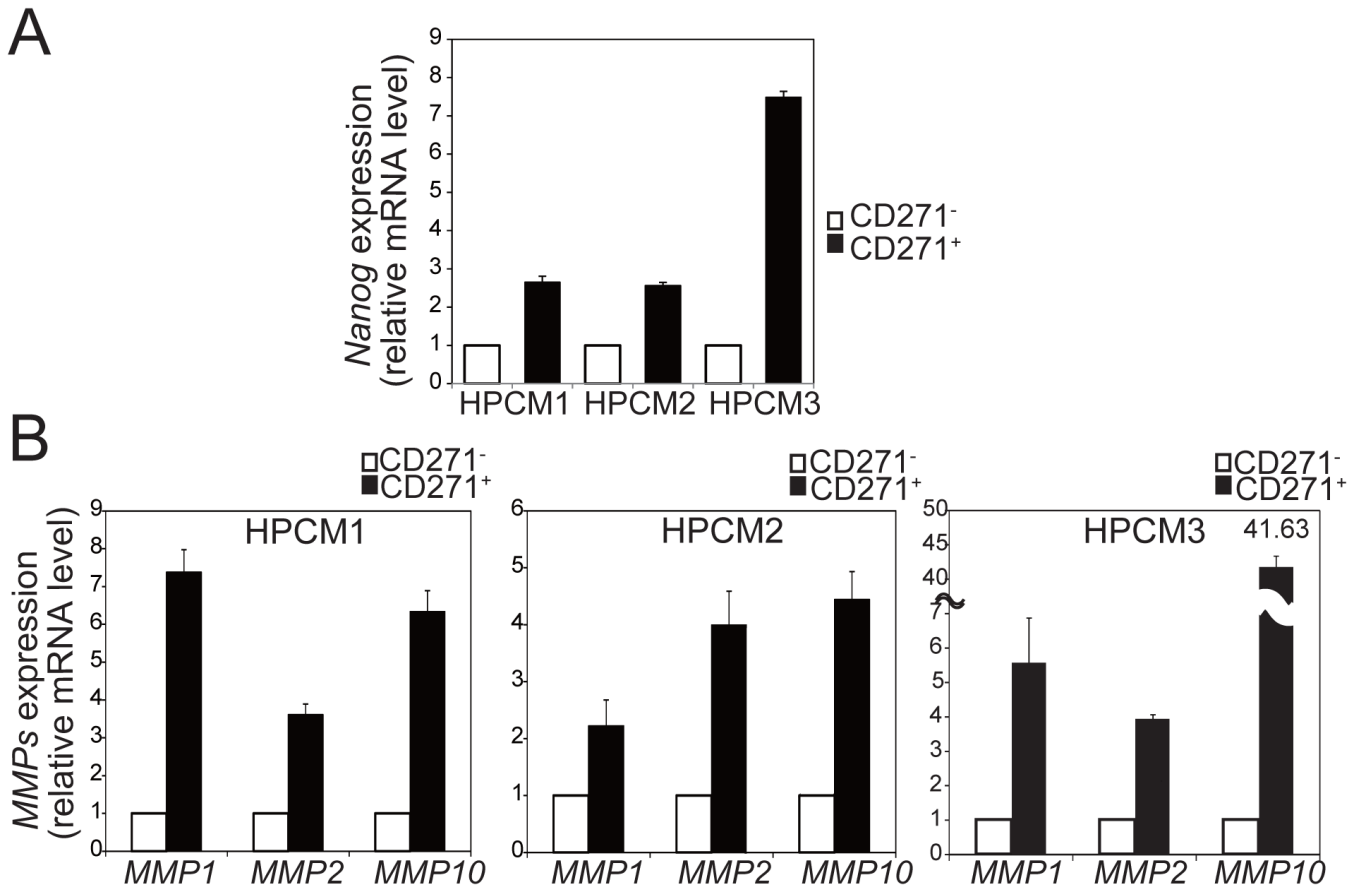
Expression profile of Nanog and MMPs in CD271^+^ and CD271^−^ cells. *Nanog* expression (A) and *MMP1*, *MMP2*, and *MMP10* expression (B) in CD271^+^ and CD271^−^ cells derived from the three HPC lines were analyzed by real-time RT-PCR. The transcript levels were normalized to those for *GAPDH*, and the fold change in the MMP expression level in CD271^+^ cells versus CD271^−^ cells was calculated for each sample. Values are the mean±SD of triplicate experiments.

We next asked whether the CD271^+^ population had characteristics associated with malignant cells in terms of stemness and invasion/metastasis. First, the expression of pluripotent stem cell-related genes, *Nanog*, *Sox2*, and *Oct-4* was examined. Real-time RT-PCR analyses indicated that the *Nanog* expression was significantly higher in the CD271^+^ cells of the three HPC lines than in the CD271^−^ cells (**Figure 3A**). IHC of serial sections showed the inclusion of CD271^+^ cells in the Nanog-positive basal layer (**Figure S3A**). However, the expression of *Sox2* and *Oct-4* showed no consistent tendency (**Figure S4**). Next, the expression of three secretion-type invasion-related genes, *MMP1*, *MMP2*, and *MMP10* was examined (**Figure 3B**). CD271^+^ cells from the three HPC lines showed a marked elevation in *MMP1*, which ranged from 2.3 to 7.3-fold compared with the CD271^−^ cells. Likewise, *MMP2* was increased 3.6–4 fold in the CD271^+^ cells. Prominent *MMP10* up-regulation was seen in the CD271^+^ population of HPCM3, at a level that was more than 40 times greater than in the CD271^−^ cells. We then performed serial IHC staining of MMP1, MMP2, and MMP10, and found that the CD271^+^ cells were positive for these MMPs (**Figure S3B**). The mRNA levels for *MMP9, MMP11, and MMP14 (MT1-MMP)* varied among the cell lines, with two lines out of the three displaying increased expressions of each MMPs (**Figure S5**). Considering the localization of CD271^+^ cells at invasive fronts, these results suggested that the CD271^+^ cells are armed with MMPs that enable tissue invasion by breaking down the extracellular matrix.

### CD271^+^ Cells are Chemoresistant *in vivo*

**Figure 4:**
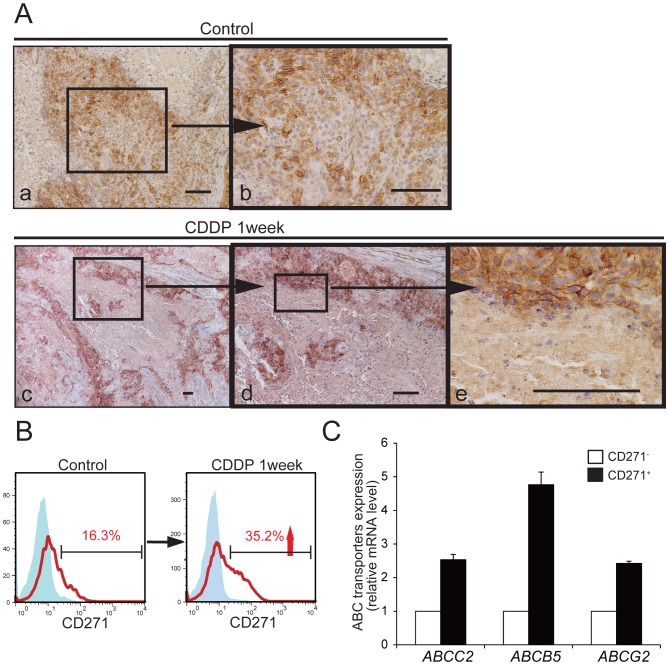
In vivo Chemoresistance of CD271^+^ cells. (A) HPCM1- transplanted mice were treated with 7.5 mg/kg of CDDP for a week, then the generated tumors were analyzed by IHC for CD271. Boxed areas are linked to their respective high-magnification images by horizontal arrows. Scale bar: 100 µm. (B) FACS analyses for CD271 in HPCM1 cells derived from mice with or without CDDP treatment. (C) Expression of *ABCC2*, *ABCB5*, and *ABCG2* in CD271^+^ and CD271^−^ cells analyzed by real-time RT-PCR. Transcript levels were normalized to those of *GAPDH*, and the fold increase of each gene expression levels in CD271^+^ cells versus CD271^−^ cells are shown. Values are the mean±SD of triplicate experiments.

To determine whether the CD271^+^ population is chemoresistant, the effect of CDDP on the cells was analyzed. Our initial *in vitro* analysis failed, due to difficulty in tissue culture maintenance, either under regular tissue culture conditions with serum or under serum-free sphere culture conditions (data not shown). Therefore, we used an *in vivo* evaluation model. Mice bearing HPCM1-derived tumors were treated with CDDP, and on day 7, the tumors were resected, sectioned, and examined by IHC for CD271 (**Figure 4A**). The flattened and keratinized CD271^−^ cells in the central zone of the tumor were in the process of dying, suggesting that chemotherapy had caused massive necrosis in these cells (**Figure 4A**). In contrast, as was most apparent in the basal layer, CD271^+^ cells survived and were surrounded by stroma. To verify that the CD271^+^ cells were refractory to CDDP, a single-cell suspension prepared from a CDDP-treated tumor was analyzed by FACS (**Figure 4B**). After CDDP administration, the CD271^+^ population increased from 16.3% to 35.2%, suggesting that the CD271^+^ cells were resistant to CDDP. The multi-drug resistance of CSCs is attributed to an elevated expression of ATP-binding transporters [19], for example, ABCC2 contributes to the efflux of CDDP and docetaxel, and ABCB5 and ABCG2 contribute to the efflux of 5-FU. We therefore compared the expression of these three ATP-binding transporters in the CD271^+^ and CD271^−^ cells of HPCM1 (**Figure 4C**). The expression of *ABCC2*, *ABCB5*, and *ABCG2* in the CD271^+^ cells was about 2.5-fold, 4.8-fold, and 2.4-fold higher than that in the CD271^−^ cells, respectively. Together, these results indicated that the CD271^+^ cells are chemoresistant.

### CD271 Expression in HPC Clinical Specimens is Correlated with a Poor Prognosis

**Figure 5:**
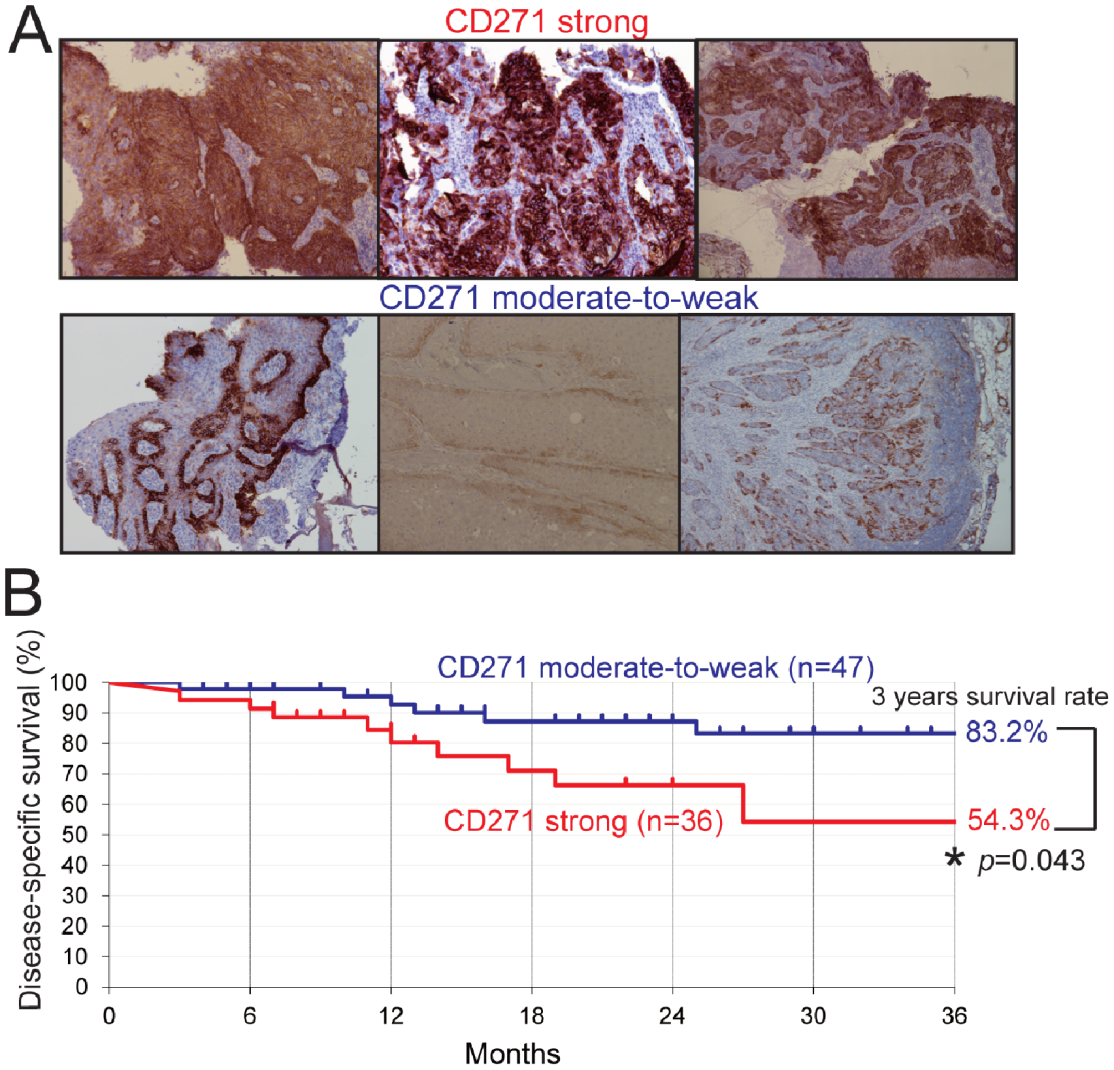
CD271 expression in HPC clinical specimens and progression of HPC patients. (A) Representative results of IHC analyses for CD271 in clinical specimens obtained from 83 HPC patients by surgery or biopsy. Immunopositivity appears brown. Specimens with more than 50% positive cells in the entire tumor were classified as “strong” (top), and less than 50%, as “moderate-to-weak” (bottom). (B) Kaplan-Meier analysis for the disease-specific survival rate (3 years) of the “strong” and “moderate-to-weak” groups. *Statistically significant.

To investigate whether CD271 expression was associated with any prognostic factors for primary HPC patients, 83 clinical specimens obtained from surgery or biopsy were examined for CD271. For this evaluation, because the CD271^+^ cells were heterogeneously located within each tumor, we used a grading system that classified each case as either “strong,” if it had more than 50% CD271^+^ cells in the entire tumor, or “moderate-to-weak,” if it had less than 50%. All the slides were evaluated independently by two investigators (I. S. and K. M.) without any prior knowledge of each patient’s clinical information. By IHC staining of the HPC tissue specimens with an anti-CD271 antibody, 36 of the 83 cases were classified as strong, and 47 were moderate-to-weak (**Figure 5A**). The clinical and pathological characteristics of the 83 patients are summarized in **Table S3**. The CD271 grade was analyzed statistically with respect to the clinical T-stage, N-stage, and the stage of disease. The percentages of cancers at the advanced T stage (T3 or more) and advanced N stage (N2 or more) were significantly higher in the CD271 strong group than in the moderate-to-weak group (47.2% vs 23.4%: *p* = 0.023, and 69.4% vs 40.4%: *p* = 0.009, respectively). Similarly, cases of advanced disease (stage III and IV) were more frequent in the CD271 strong group than in the moderate-to-weak group (80.6% vs 55.3%: *p* = 0.016). Next, the disease-specific survival rate was compared using Kaplan-Meier methods. The three-year survival rate was significantly lower in the CD271 strong group compared to the CD271 moderate-to-weak group (54.3% vs 83.2%: *p* = 0.043) (**Figure 5B**). These data indicate that the histological expression of CD271 is associated with poor clinical outcomes.

**Figure 6:**
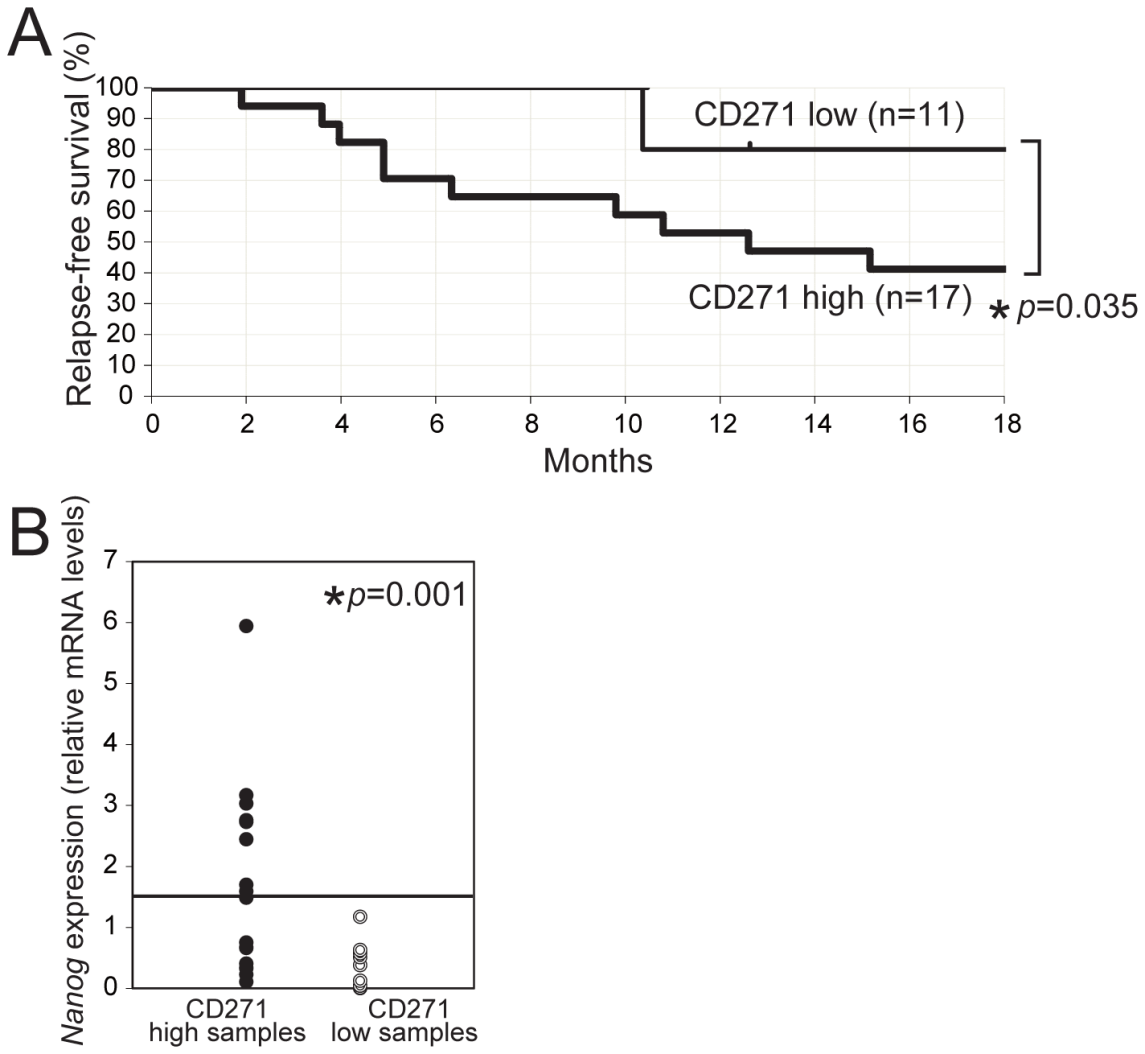
Gene expression of CD271 and Nanog in clinical specimens of HPC. (A) Kaplan-Meier analysis for the relapse-free survival rate according to the *CD271* mRNA level, in tumor tissues derived from 28 HPC patients by surgery. Real-time RT-PCR analyses were conducted, normalized to the expression of *GAPDH*, and the fold-change in *CD271* expression levels in the tumor versus normal mucosa was calculated. The cut-off value for the *CD271*-expression index was 1.5. *Statistically significant. (B) *Nanog* expression was examined by real-time RT-PCR. Cut-off value for the *Nanog* expression index was 1.5. *Statistically significant. Closed circles, CD271 high; open circles, CD271 low.

We further examined whether the *CD271* gene expression in clinical specimens was associated with patients’ prognosis. For this analysis, 28 cases of HPC that were completely resected, as assessed both surgically and histologically, were examined for *CD271* expression. Immediately after resection, each specimen was separated into tumor and normal hypopharyngeal mucosa. The total RNAs purified from these respective tissues, were subjected to *CD271* mRNA quantification. Because normal mucosa also expresses CD271 (**Figure 1C**), we used a “CD271-expression index,” the value of *CD271* in tumor versus that in normal mucosa, as the CD271 level. During the average 20-month follow-up period, 11 of the 28 cases relapsed. All of the relapses occurred within 18 months, and the relapse-free survival for 18 months was analyzed using Kaplan-Meier methods (**Figure 6A**). Using a cut-off value for the CD271-expression index of 1.5, the *CD271*-high expressers relapsed significantly more frequently than the *CD271*-low cases (41.1% vs 80.0% relapse-free survival, respectively: *p* = 0.035). The demographic and clinical characteristics of these patients are shown in **Table S4**. For the clinical disease classification, pT3 or more was significantly more frequent in the CD271-high cases (CD271-high 15/17, CD271-low 5/11; *p* = 0.022). Taken together, these results indicated that CD271 expression is associated with poor clinical outcomes in terms of prognosis and relapse.

### Nanog Expression is Associated with CD271 High Tumors

Next, because the *CD271* expression in HPC tumors had a positive relationship with that of *Nanog*, the *Nanog* expression in the same set of 28 clinical samples was quantified and analyzed. There was a significant association between the *CD271*-expression index and the *Nanog* index, which was the level of *Nanog* mRNA in the tumor versus that in normal mucosa (**Figure 6B**). Using a cut-off score 1.5 for the *Nanog* index, all the *Nanog*-high expressers (8 cases) were categorized into the *CD271*-high group. Taken together, these results suggest that both CD271 and *Nanog* are strong prognostic factors for HPC.

## Discussion

The identification of cell-surface markers to define CSCs/CICs is important for the possible establishment of target-specific therapies using small molecule inhibitors and/or humanized antibodies. In this report, we show that the CD271^+^ cells in HPC possess tumor-initiating capability *in vivo*. The CD271^+^ cells are endowed with several CSC-like characteristics, including 1) tumor initiation, 2) high expression of the CSC-related gene *Nanog*, 3) self-renewal and the capacity to generate hierarchical populations, 4) chemoresistance, and 5) invasion capacity. Furthermore, we demonstrated that the expression of CD271 in HPC tumors is associated with a poor prognosis for HPC patients.

CSCs were first identified in acute myeloid leukemia (AML). A small subpopulation of AML cells with the CD34^+^CD38^−^ phenotype was observed to initiate tumors in immunodeficient mice [20]. Since then, xenograft models using highly immunodeficient mice have become widely used to evaluate the cancer stem cells within clinical cancer specimens, and xenotransplantation is currently regarded as the “gold standard” in this field [21]. Using this assay system, a number of solid tumors have been analyzed. Breast cancer cells with CD44^+^CD24^−^ were identified as CSCs, which form heterogeneous tumors with CD44^+/−^ and CD24^+/−^ phylogeny [22]. For HNSCC, only two reports have analyzed clinical specimen-based CSCs using xenotransplantation. Prince et al demonstrated that 5,000 CD44^+^ cells could initiate a tumor [7], whereas Clay et al. demonstrated that 500 ALDH^+^ cells formed a tumor [23]. Because positivity for ALDH is based on its enzyme activity, CD44 has been the cell-surface CSC marker studied in most detail. The same xenotransplantation methodology led to the characterization of CD133 as a laryngeal cancer stem cell marker using Hep-2 cells [24]. However, the CD133^+^ cells from clinical HNSCC specimens have not been analyzed by xenotransplantation.

In our three primary HPC cases, CD133 expression was negative, as judged by FACS analyses and IHC (data not shown). Instead, we compared two independent subpopulations, CD44^+^ and CD44^−^. However, no significant difference was observed in the tumor-initiating capability of these populations *in vivo*; a minimum of 100 cells from either population initiated tumors (**Table S5**). Furthermore, a recent report showed that both the CD44^−^ and CD44^+^ cells of clinical HNSCC specimens possess similar sphere-forming and tumor-initiating capabilities, as well as chemoresistance [8]. Our results are consistent with this notion, with regard to the tumor-initiating capability.

Although categorized as HNSCC, HPC may have independent characteristics from other HNSCCs, especially with regard to the CSCs. Indeed, CSC markers are divergent, depending on the type of cancer. For example, CD44^+^CD24^−^ indicates breast cancer stem cells [22], whereas a pancreatic cancer stem cell phenotype is CD44^+^CD24^+^ [25], and CD44^+^CD117^+^ is an ovarian cancer stem cell phenotype [26]. Breast cancers, for instance, are classified into several subtypes, such as luminal, basal, and HER2^+^, but the well-known CD44^+^CD24^−^ phenotype is closely associated with the basal type, and the HER2^+^-type CSCs show an ALDH^+^ phenotype [27]. Thus, since each cancer expresses a unique pattern of CSC markers, the CSC markers may also differ among types of HNSCCs.

In a variety of cancers, the CSCs are considered to be derived from the stem cells of normal tissues or from cancer cells acquiring stem cell pluripotency by accumulating genetic mutations [6]. Previous studies indicated that CD271 is expressed in tissue stem cells. In normal oral [11], laryngeal [12], and esophageal mucosa [13], CD271^+^ cells are distributed in the basal layer. These observations are consistent with our present finding that CD271^+^ cells were distributed in the basal layer of normal hypopharyngeal mucosa. Based on the hypothesis that CD271 is a tissue stem cell marker of the head and neck region, it is tempting to speculate that HPC CSCs are derived from the stem cells of normal mucosa.

In the present study, we observed that the CD271^+^ cells of HPC possessed several characteristics of CSCs. First, the CD271^+^ expression ranged from 2 to 20% of the tumor cells. Second, CD271^+^ cells were more tumorigenic than CD271^−^ cells. Third, the CD271^+^ cells generated tumors that showed a striking similarity to the original ones *in vivo*, in which a heterogeneous population of CD271^+^ and CD271^−^ cells was present. These results indicated that the CD271^+^ CSCs of HPC might originate from normal stem cells of the hypopharyngeal mucosa. Accordingly, recent reports propose that CD271 is a CSC marker of squamous cell carcinomas of esophageal origin [16] as well as malignant melanoma [14], [15].

Accumulating evidence suggests that some CSCs reside close to blood vessels, in the perivascular niche [28], [29]. In this regard, Krishnamurthy et al. demonstrated that the ALDH^+^ cells of HNSCC are located within a 100-µm radius of blood vessels. The selective ablation of endothelial cells reduced the fraction of CD44^+^ALDH^+^ cells, suggesting that endothelial cells may form a microenvironment, or niche, that allows CSC survival and self-renewal [30]. In our analyses, the localization of CD271^+^ HPC cells indicated that they might have been in direct contact with blood vessels, suggesting that either direct cell-to-cell interaction or some paracrine factor may promote the survival of CSCs. One interesting possibility is neurotrophins such as NGF support CD271^+^ cells. The perivascular niche hypothesis needs to be further investigated by examining the CD271^+^ cells’ fate in HPC.

We also observed in the current study that CD271^+^ cells were clustered within the invasive front area. The invasive front is the part of a growing tumor that extends most deeply into the adjacent non-cancerous tissue, and it is the location of the tumor-host communication interface [31]–[33]. Histopathological grading analyses revealed that the invasive front reflects the biological aggressiveness of a carcinoma [33]. In some of our specimens, CD271^+^ cells formed small foci, which might be potential points of invasion initiation (**Figure 1C** **–c,d**). In accordance with this finding, the expressions of *MMP1*, *MMP2*, and *MMP10* were elevated in the CD271^+^ cells. Because these secreted *MMPs* cleave the extracellular matrix, they are essential factors for invasion [31], [34]. MMPs have been shown to contribute to tumorigenicity; the over-expression of *MMP1* enhances tumorigenicity, while its knockdown reduces tumor formation in a glioblastoma cell line [35]. In non-small cell lung cancers, MMP10 plays a pertinent role in tumor initiation, and it maintains the characteristics of CSCs [36]. Considering these reports, it is reasonable that MMP-positive CD271^+^ cells show strong invasion capability as well as the potential to initiate tumors.

Nanog is one of the homeobox transcription factors expressed in pluripotent embryonic stem cells [37], [38], and accumulating evidence suggests it has a role in inducing the pluripotency of various types of cancer. The over-expression of *Nanog* leads to elevated tumorigenicity [39], [40], while its inhibition reduces tumorigenicity in prostate, breast, and colorectal cancers [39], [41]. In HNSCC, sphere culture enhances the expression of *Nanog*, and in clinically dissected samples, high *Nanog* expression is closely associated with a poor prognosis for the patient [42]. In our study, the *Nanog* expression was elevated in the CD271^+^ cells of all three HPC xenograft lines. Furthermore, all the *Nanog*-high cases also had *CD271*-high expression in our clinical specimens. Thus, we consider the CD271^+^ cells in HPC to have pluripotent stemness characteristics with high *Nanog* expression.

Treatment resistance is a well-known characteristic of CSCs. For example, CD133^+^ glioma stem cells show radioresistance [43], and CD44^+^CD24^−^ breast CSCs are chemoresistant [44]. These treatment resistances are considered to be associated with treatment failure, for instance with cancer relapse. In our study, the CD271^+^ cells of HPC were at least partially CDDP-resistant as assessed by both IHC and FACS analyses. In esophageal SCC, Huang et al previously demonstrated a similar CDDP resistance of CD271^+^ cells in vitro using ^64^copper accumulation assays [16]. Our findings also suggested the involvement of the drug transporters ABCC2, ABCB5, and ABCG2 in the function of the CD271^+^ cells. The elevated *ABCC2* expression may be responsible for the CDDP resistance of the CD271^+^ cells [45]. The up-regulation of *ABCB5* and *ABCG2* may similarly confer multidrug resistance on CD271^+^ cells. Interestingly, ABCB5 marks a putative cancer stem cell compartment in oral cancer [46], and cancer stem cells of the laryngeal cancer cell line Hep-2 show an increase in *ABCG2* expression [47]. Taken together, the up-regulation of ATP-binding transporters may contribute to the malignant phenotypes of CD271^+^ cells. However, the differential drug resistance *in vivo* needs to be interpreted carefully, because additional factors, such as differential localization within a tumor and interaction with the stroma may affect cell survival. In the present study, treatment resistance was also indirectly supported by the clinical outcomes. Immunohistochemical and gene expression analyses for CD271 using primary HPC specimens indicated that high CD271 expression was correlated with a poor prognosis for the patient. Disease-specific survival analyses of 83 HPC cases, as well as the relapse-free survival, suggested that the CD271 expression determined by IHC staining was correlated with a poor prognosis and tumor aggressiveness. From these findings, we speculate that elevated CD271 expression is a good marker for a poor prognosis. Similarly, CD271 is reported to be a marker for poor prognosis in oral cancer [48]. Accordingly, we are in the process of setting up a larger clinico-pathological study to investigate whether CD271 can be a unique prognostic marker of HPC, when adjusting multiple parameters, such as clinical-, T- and N-stages. At present, with limited number of cases, both the disease-specific and relapse-free survival rates of CD271 high cases tend to be lower (e.g., Stage IV, 18-month relapse-free survival: CD271-low, 66.7% vs CD271-high, 18.2%, *p* = 0.09). By increasing the number of cases, we speculate that CD271 can be an independent prognostic marker of HPC in the future.

There are some limitations in our study. First, despite the enhanced tumor-initiating capability of the CD271^+^ cells, the CD271^−^ cells also initiated tumors, but with a longer latency and less efficiency. Furthermore, IHC and FACS analyses of the CD271^–^derived tumors revealed that they included some CD271^+^ cells (**Figure S6**). A certain level of plasticity may account for this inter-conversion. Alternatively, a minor contamination of the CD271^−^ cells by CD271^+^ cells during the sorting procedure cannot be ruled out. Second, the subcutaneous xenografting experiment requires careful evaluation, as it may differ from orthotopic grafting. In addition, CD271-positivity detected by IHC and FACS may potentially include certain difference in the sensitivity, the dynamic range, and the specificity. Therefore, careful interpretation may be required upon comparison of the data obtained by these two methods.

Taken together, we conclude that the CD271^+^ cells in human HPC possess tumor-initiating capability and some CSC-like characteristics. Our findings will help pave the way for the development of a novel strategy for treating HPC, using CD271, for future clinical interventions. Further study will be needed to investigate the function of CD271 in CSCs.

## Supporting Information

Figure S1 **Histology of xenotransplanted HPC tumors and their original primary samples.** Primary HPC tumors obtained from three independent patients were transplanted into NOG mice, and the respective xenotransplanted tumors were dissected from the mice. Tissues of the primary HPC tumors and their xenotransplanted tumors were stained with hematoxylin and eosin (H&E). The primary and xenotransplanted tumors were histologically indistinguishable. Scale bar: 100 µm.
(TIF)

Figure S2 **IHC of xenotransplanted HPC tumors for CD271 and CKs.** IHC for CD271, CK5/6, and CK8 in serial sections of a xenograft tumor. IHC is performed as described in Materials and Methods S1. Immunopositivity appears brown. Scale bar: 100 µm.
(TIF)

Figure S3 **IHC of xenotransplanted HPC tumors for Nanog and MMPs.** IHC for CD271 and Nanog (A), and CD271 and MMPs (B) in serial sections of a xenograft tumor. IHC is performed as described in Materials and Methods S1. Immunopositivity appears brown. Scale bar: 100 µm.
(TIF)

Figure S4 **Expression of Sox-2 and Oct-4 in CD271^+^ and CD271^−^ cells from HPC.** *Sox-2* and *Oct-4* expression in the CD271^+^ and CD271^−^ cells was analyzed by real-time RT-PCR. Transcript levels were normalized to that of *GAPDH*, and the fold increase in the expression level in CD271^+^ versus CD271^−^ cells was calculated for each HPC line. Values are the mean±SD of triplicate experiments.
(TIF)

Figure S5 **Expression of** ***MMP9*** **,** ***MMP11*** **, and** ***MT1-MMP*** **in the CD271^+^ and CD271** ^−^ **cells of HPC.** The *MMP9*, *MMP11*, and *MT1-MMP* expressions in CD271^+^ and CD271^−^ cells were analyzed by real-time RT-PCR. The transcript levels were normalized to that of *GAPDH*, and the fold change in *MMP9*, *MMP11* and *MT1-MMP* expression levels in CD271^+^ versus CD271^−^ cells was calculated for each sample. Values are the mean±SD of triplicate experiments.
(TIF)

Figure S6 **Plasticity between the CD271^−^ and CD271^+^ populations.** Tumors generated from CD271^−^ cells, and CD271^+^ cells were analyzed by IHC and FACS for CD271. Immunopositivity appears brown. Scale bar: 100 µm.
(TIF)

Table S1 **Primer Sequence.**
(DOCX)

Table S2 **Short summary of HPC xenograft lines.**
(DOCX)

Table S3 **Correlation between CD271 expression in IHC and characteristics of HPC patients.**
(DOCX)

Table S4 **Correlation between CD271 expression and clinical characteristics of HPC patients.**
(DOCX)

Table S5 **Tumorigenicity of CD44^+^ and CD44^−^ cells in vivo (HPCM1).** *In vivo* tumorigenesis assay is performed as described in Materials and Methods S1.
(DOCX)

Materials and Methods S1. (DOCX)
