# Supplementary figures and images for: CD271 Defines a Stem Cell-Like Population in Hypopharyngeal Cancer
Source: PLoS One. 2013 Apr 23;8(4):e62002. doi: 10.1371/journal.pone.0062002 (PMC3633921; doi:10.1371/journal.pone.0062002)

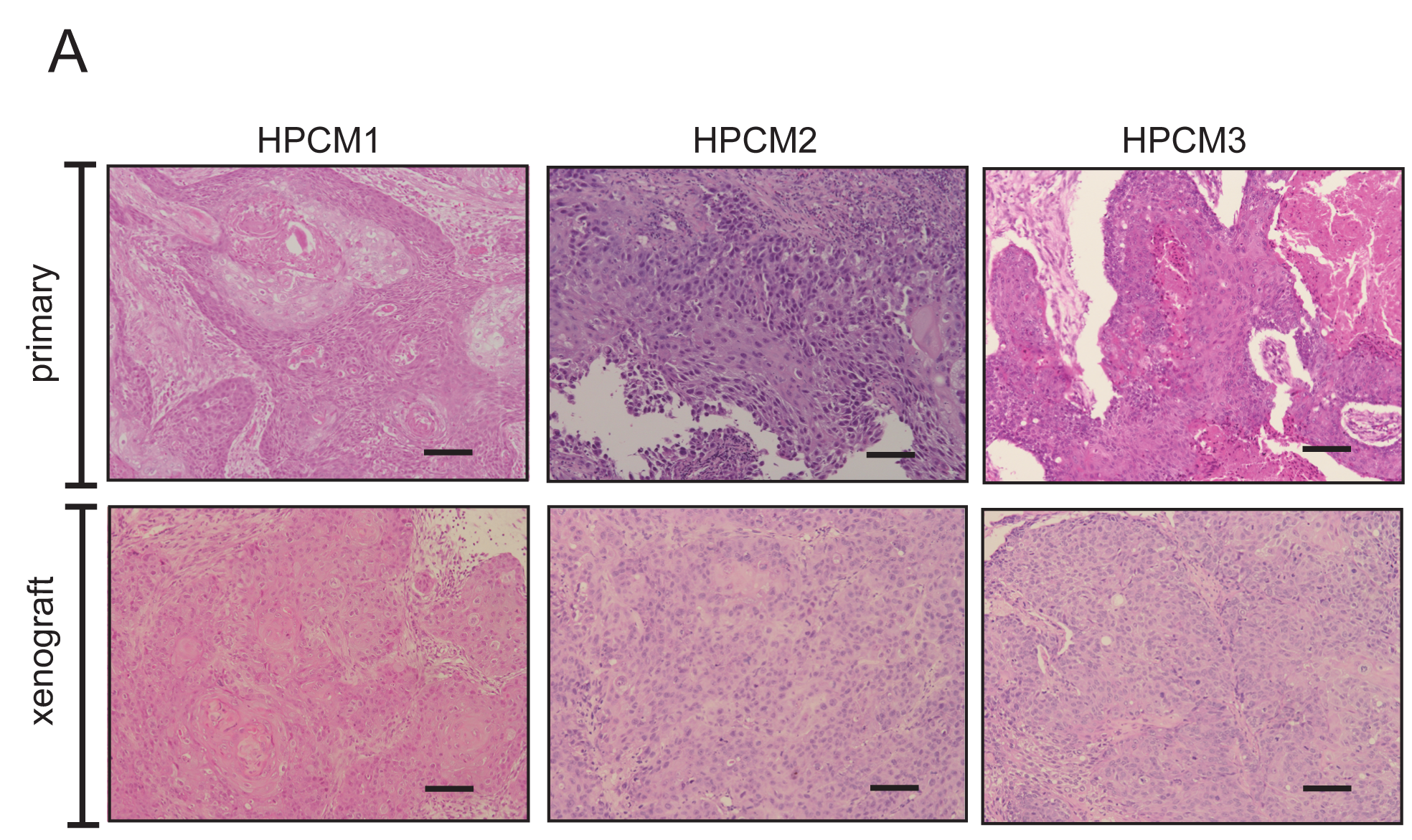

Supplement: Figure S1 — Histology of xenotransplanted HPC tumors and their original primary samples. Primary HPC tumors obtained from three independent patients were transplanted into NOG mice, and the respective xenotransplanted tumors were dissected from the mice. Tissues of the primary HPC tumors and their xenotransplanted tumors were stained with hematoxylin and eosin (H&E). The primary and xenotransplanted tumors were histologically indistinguishable. Scale bar: 100 µm. (TIF) [file pone.0062002.s001.tif]

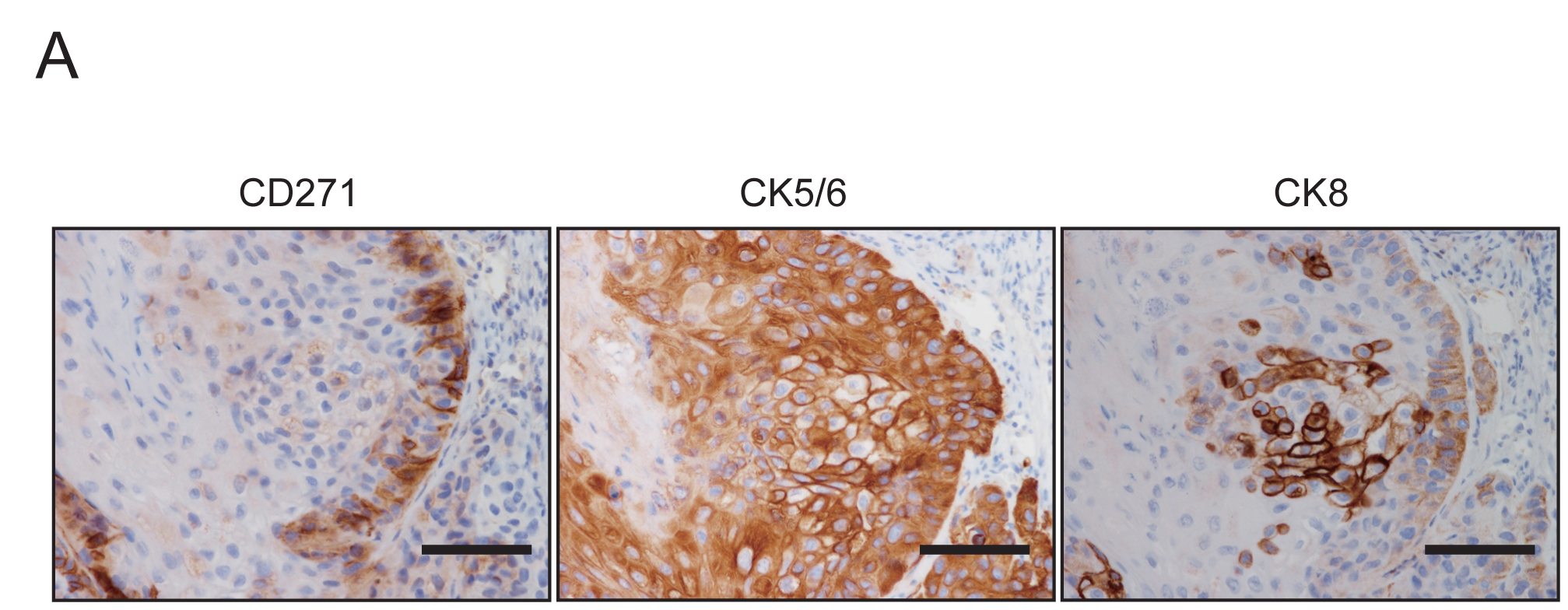

Supplement: Figure S2 — IHC of xenotransplanted HPC tumors for CD271 and CKs. IHC for CD271, CK5/6, and CK8 in serial sections of a xenograft tumor. IHC is performed as described in Materials and Methods S1. Immunopositivity appears brown. Scale bar: 100 µm. (TIF) [file pone.0062002.s002.tif]

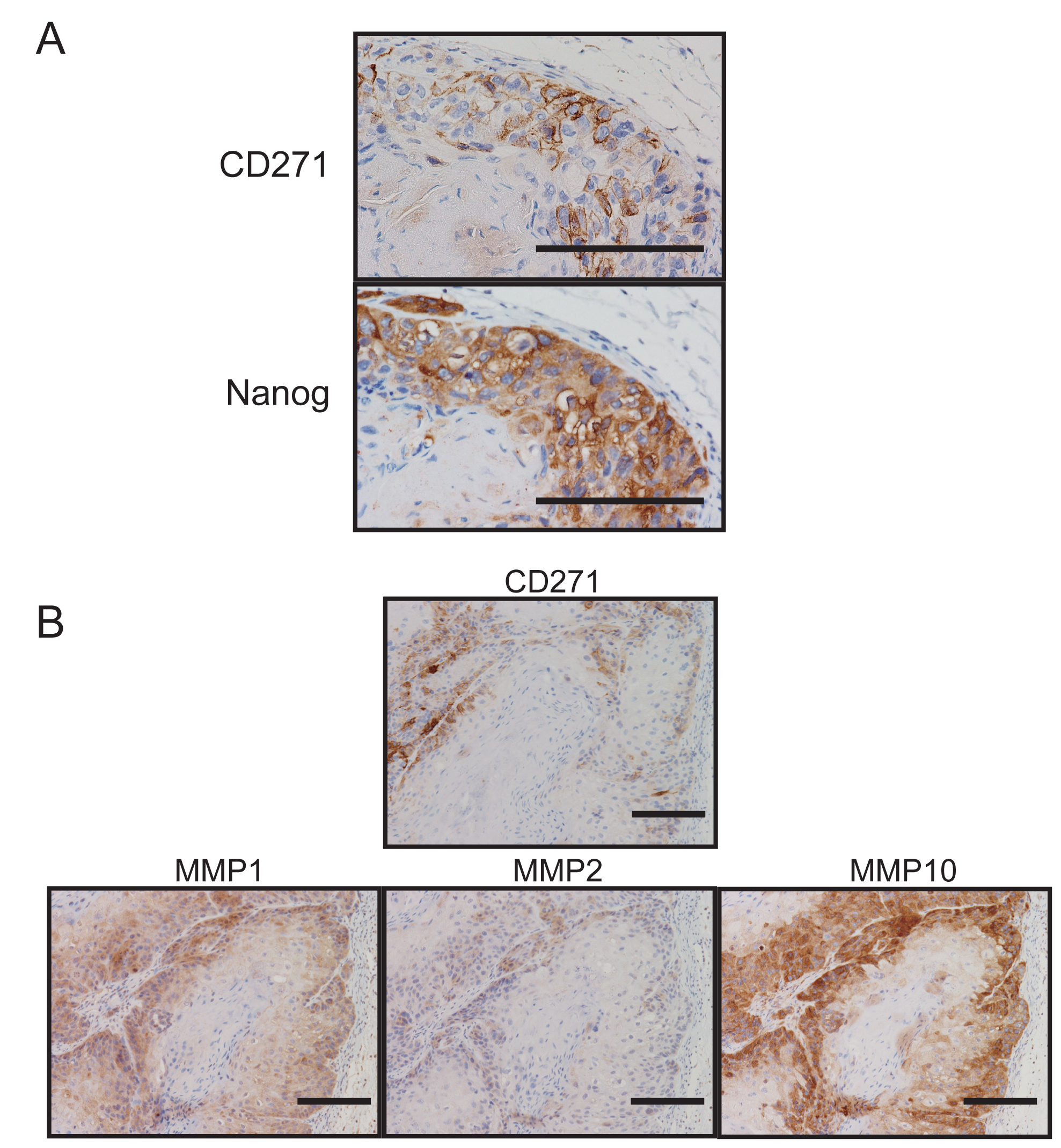

Supplement: Figure S3 — IHC of xenotransplanted HPC tumors for Nanog and MMPs. IHC for CD271 and Nanog (A), and CD271 and MMPs (B) in serial sections of a xenograft tumor. IHC is performed as described in Materials and Methods S1. Immunopositivity appears brown. Scale bar: 100 µm. (TIF) [file pone.0062002.s003.tif]

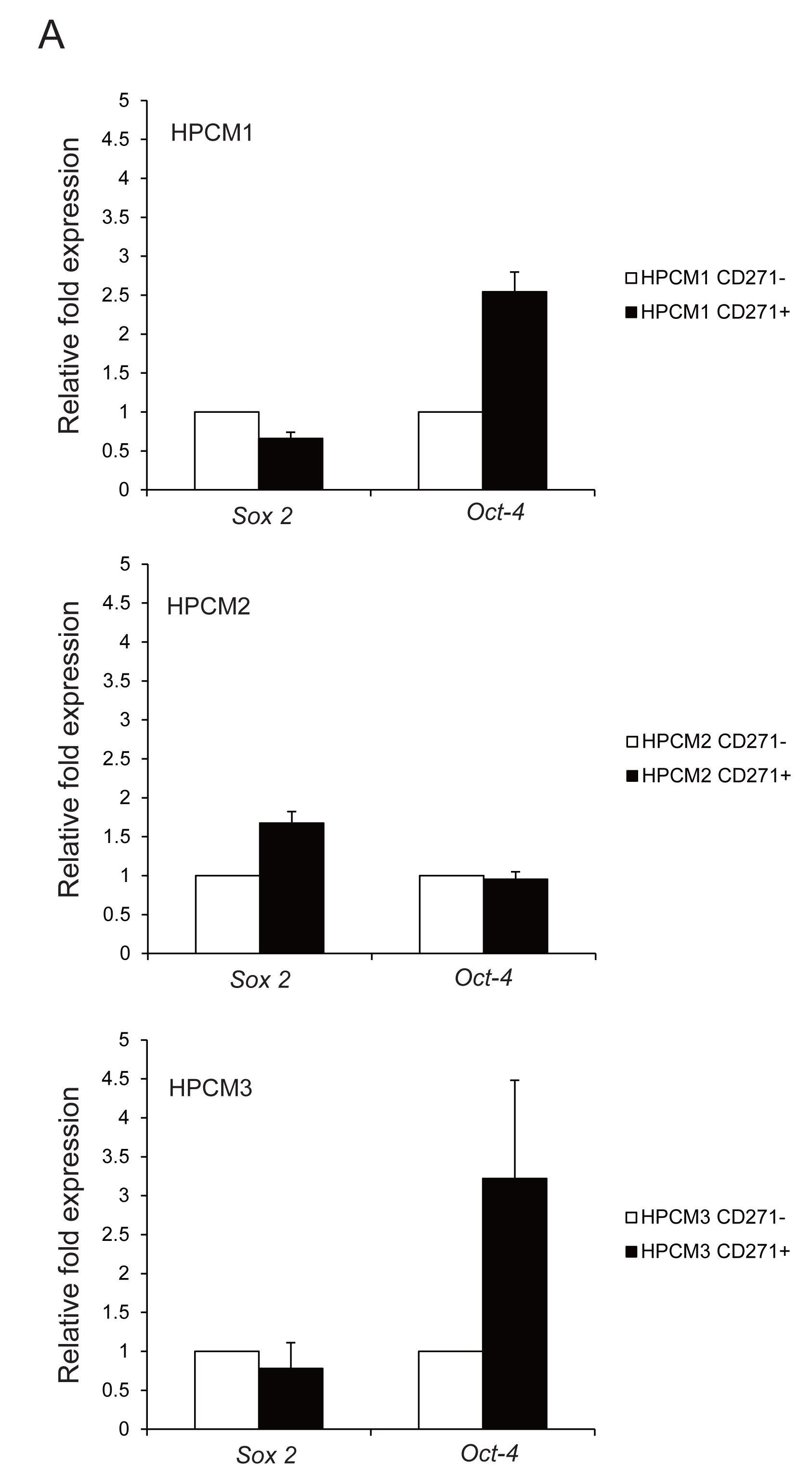

Supplement: Figure S4 — Expression of Sox-2 and Oct-4 in CD271+ and CD271− cells from HPC. Sox-2 and Oct-4 expression in the CD271+ and CD271− cells was analyzed by real-time RT-PCR. Transcript levels were normalized to that of GAPDH, and the fold increase in the expression level in CD271+ versus CD271− cells was calculated for each HPC line. Values are the mean±SD of triplicate experiments. (TIF) [file pone.0062002.s004.tif]

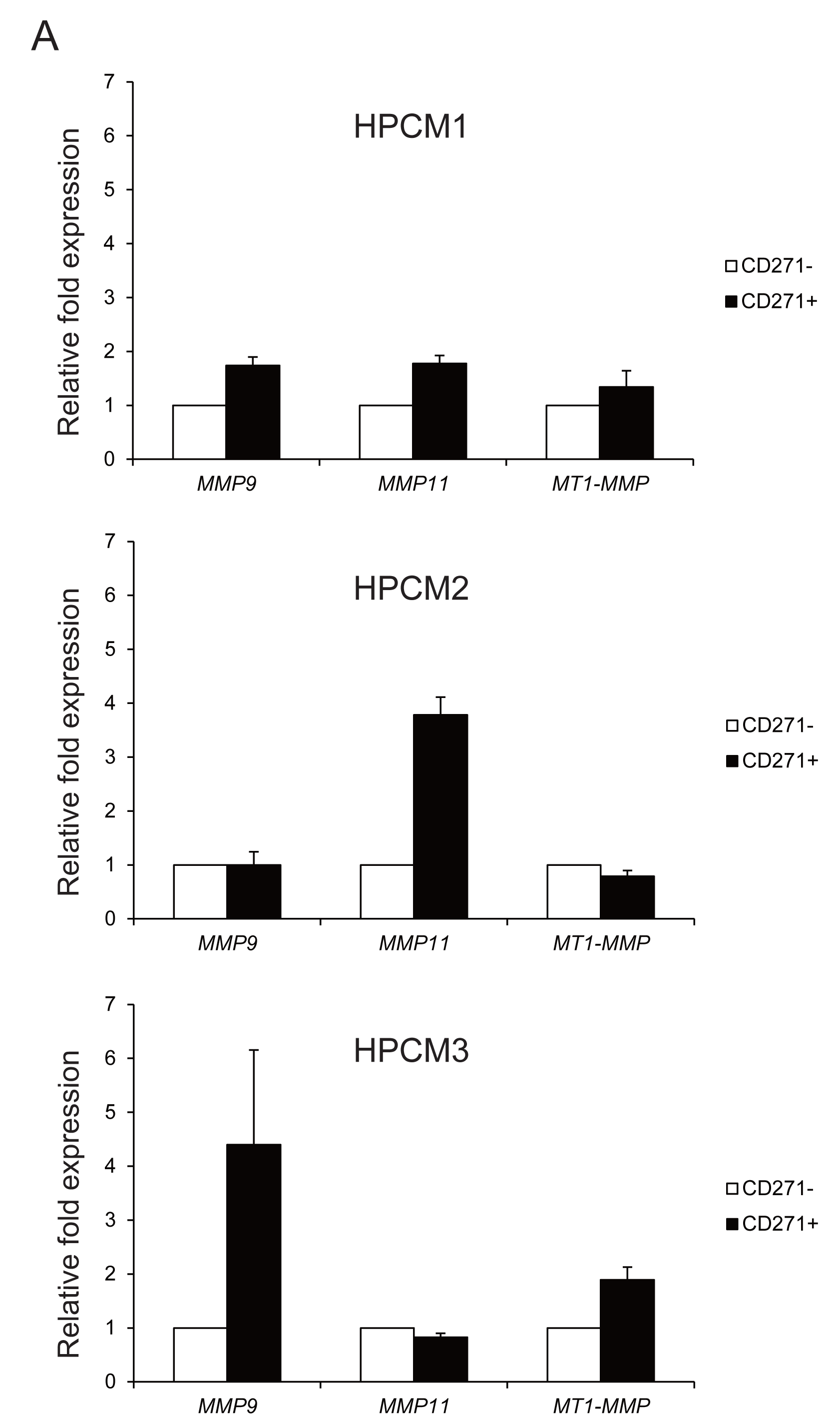

Supplement: Figure S5 — Expression of MMP9 , MMP11 , and MT1-MMP in the CD271+ and CD271 − cells of HPC. The MMP9, MMP11, and MT1-MMP expressions in CD271+ and CD271− cells were analyzed by real-time RT-PCR. The transcript levels were normalized to that of GAPDH, and the fold change in MMP9, MMP11 and MT1-MMP expression levels in CD271+ versus CD271− cells was calculated for each sample. Values are the mean±SD of triplicate experiments. (TIF) [file pone.0062002.s005.tif]

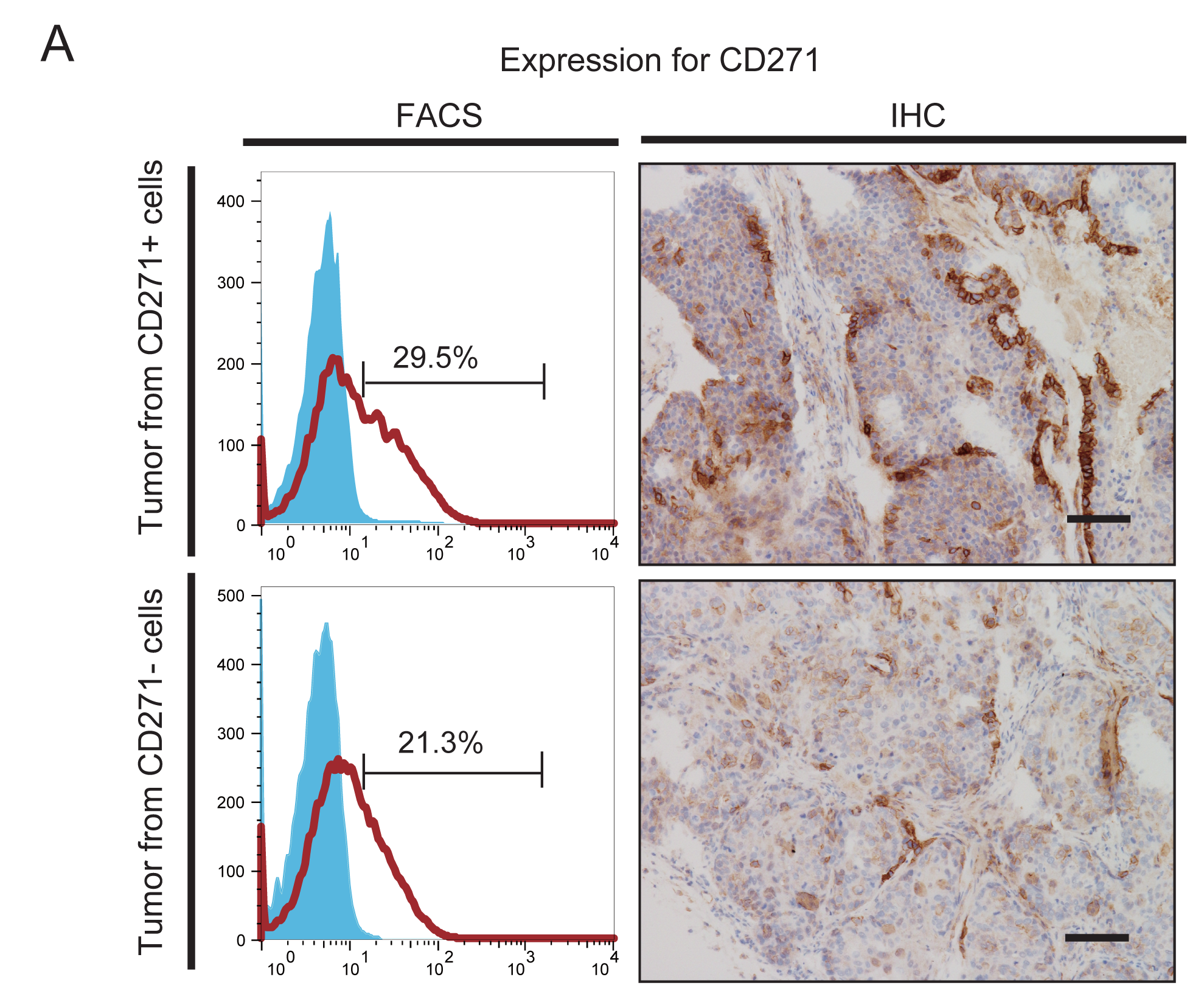

Supplement: Figure S6 — Plasticity between the CD271− and CD271+ populations. Tumors generated from CD271− cells, and CD271+ cells were analyzed by IHC and FACS for CD271. Immunopositivity appears brown. Scale bar: 100 µm. (TIF) [file pone.0062002.s006.tif]
