# Supplementary material for: CD271 Defines a Stem Cell-Like Population in Hypopharyngeal Cancer
Source: PLoS One. 2013 Apr 23;8(4):e62002. doi: 10.1371/journal.pone.0062002 (PMC3633921; doi:10.1371/journal.pone.0062002)
Supplement: Table S1 — Primer Sequence. (DOCX) [file pone.0062002.s007.docx]

**Table S1**. Primer Sequence.

| Gene | F/R | Primer sequence | Product size (bp) |
| --- | --- | --- | --- |
| *CD271* | Forward | AACCTCATCCCTGTCTATTG | 111 |
|  | Reverse | GTTGGCTCCTTGCTTGTT |  |
| *GAPDH* | Forward | TGAAGGTCGGAGTCAACGG | 65 |
|  | Reverse | AGAGTTAAAAGCAGCCCTGGTG |  |
| *Nanog* | Forward | ATGCCTCACACGGAGACTGT | 67 |
|  | Reverse | AGGGCTGTCCTGAATAAGCA |  |
| *Sox2* | Forward | TGGCTCCATGGGTTCGGTGG | 140 |
|  | Reverse | TGTGAAGTCTGCTGGGGGCG |  |
| *Oct-4* | Forward | GCCCCATTTTGGTACCCCAG | 101 |
|  | Reverse | ATGGGAGAGCCCAGAGTGGT |  |
| *MMP1* | Forward | GCTAACCTTTGATGCTATAACTACGA | 78 |
|  | Reverse | GGATTTGTGCGCATGTAGAA |  |
| *MMP2* | Forward | CCCCAAAACGGACAAAGAG | 87 |
|  | Reverse | CTTCAGCACAAACAGGTTGC |  |
| *MMP9* | Forward | GAACCAATCTCACCGACAGG | 67 |
|  | Reverse | GCCACCCGAGTGTAACCATA |  |
| *MMP10* | Forward | GCAAAAGAGGAGGACTCCAA | 76 |
|  | Reverse | TCACATCCTTTTCGAGGTTGTA |  |
| *MMP11* | Forward | GGTGCCCTCTGAGATCGAC | 92 |
|  | Reverse | TCACAGGGTCAAACTTCCAGT |  |
| *MT1-MMP* | Forward | GCCTTGGACTGTCAGGAATG | 104 |
|  | Reverse | AGGGGTCACTGGAATGCTC |  |
| *ABCC2* | Forward | AGTGAATGACATCTTCACGTTTG | 63 |
|  | Reverse | CTTGCAAAGGAGATCAGCAA |  |
| *ABCB5* | Forward | CACAAAAGGCCATTCAGGCT | 75 |
|  | Reverse | GCTGAGGAATCCACCCAATCT |  |
| *ABCG2* | Forward | TCAATCAAAGTGCTTCTTTTTTATG | 150 |
|  | Reverse | TTGTGGAAGAATCACGTGGC |  |
