# Supplementary material for: CD271 Defines a Stem Cell-Like Population in Hypopharyngeal Cancer
Source: PLoS One. 2013 Apr 23;8(4):e62002. doi: 10.1371/journal.pone.0062002 (PMC3633921; doi:10.1371/journal.pone.0062002)
Supplement: Table S2 — Short summary of HPC xenograft lines. (DOCX) [file pone.0062002.s008.docx]

**Table S2**. Short summary of HPC xenograft lines.

|  | T | N | M | stage | subsite | pathology |
| --- | --- | --- | --- | --- | --- | --- |
| HPCM1 | 3 | 2b | 0 | IVa | ^a^PS | SCC |
| HPCM2 | 3 | 2c | 0 | IVa | ^a^PS | SCC |
| HPCM3 | 4a | 2c | 0 | IVa | ^a^PS | SCC |

^a^PS indicates piriform sinus.
