# Supplementary material for: CD271 Defines a Stem Cell-Like Population in Hypopharyngeal Cancer
Source: PLoS One. 2013 Apr 23;8(4):e62002. doi: 10.1371/journal.pone.0062002 (PMC3633921; doi:10.1371/journal.pone.0062002)
Supplement: Table S3 — Correlation between CD271 expression in IHC and characteristics of HPC patients. (DOCX) [file pone.0062002.s009.docx]

**Table S3**. Correlation between CD271 expression in IHC and characteristics of HPC patients.

|  |  |  | **CD271 expression** | |  |  |
| --- | --- | --- | --- | --- | --- | --- |
|  |  | **No. of cases** | **high** | **moderate to weak** | ***p* value** | |
| **Total** | |  |  |  |  |  |
|  |  | 83 | 36 | 47 |  |  |
| **T** | |  |  |  |  |  |
|  | T4 | 16 | 10 | 6 | 0.086 | (≧T4) |
|  | T3 | 12 | 7 | 5 | ^a^0.023 | (≧T3) |
|  | T2 | 37 | 15 | 22 | ^a^0.041 | (≧T2) |
|  | T1 | 18 | 4 | 14 |  |  |
| **N** | |  |  |  |  |  |
|  | N3 | 3 | 1 | 2 | 0.721 | (≧N3) |
|  | N2 | 41 | 24 | 17 | ^a^0.009 | (≧N2) |
|  | N1 | 6 | 1 | 5 | 0.051 | (≧N1) |
|  | N0 | 33 | 10 | 23 |  |  |
| **Stage** | |  |  |  |  |  |
|  | IV | 43 | 25 | 18 | ^a^0.005 | (≧IV) |
|  | III | 12 | 4 | 8 | ^a^0.016 | (≧III) |
|  | II | 18 | 5 | 13 | 0.112 | (≧II) |
|  | I | 10 | 2 | 8 |  |  |
| **Sex** | |  |  |  |  |  |
|  | male | 81 | 36 | 45 | 0.21 |  |
|  | female | 2 | 0 | 2 |  |  |
| **Age** | |  |  |  |  |  |
|  | average | 67.1 | 70.8 | 64.2 | ^a^0.0003 |  |
|  | median | 67 | 70 | 64 |  |  |

^a^Statistically significant.
