# Supplementary material for: CD271 Defines a Stem Cell-Like Population in Hypopharyngeal Cancer
Source: PLoS One. 2013 Apr 23;8(4):e62002. doi: 10.1371/journal.pone.0062002 (PMC3633921; doi:10.1371/journal.pone.0062002)
Supplement: Table S4 — Correlation between CD271 expression and clinical characteristics of HPC patients. (DOCX) [file pone.0062002.s010.docx]

**Table S4**. Correlation between CD271 expression and clinical characteristics of HPC patients.

|  |  |  | ***CD271* expression** | |  |  |
| --- | --- | --- | --- | --- | --- | --- |
|  |  | **No. of cases** | **high** | **low** | ***p* value** | |
| **Total** | |  |  |  |  |  |
|  |  | 28 | 17 | 11 |  |  |
| **pT** | |  |  |  |  |  |
|  | pT4 | 9 | 7 | 2 | 0.197 | (≧pT4) |
|  | pT3 | 11 | 8 | 3 | ^a^0.022 | (≧pT3) |
|  | pT2 | 8 | 2 | 6 | none | (≧pT2) |
|  | pT1 | 0 | 0 | 0 | none |  |
| **pN** | |  |  |  |  |  |
|  | pN3 | 0 | 0 | 0 | none | (≧pN3) |
|  | pN2 | 17 | 10 | 7 | 0.558 | (≧pN2) |
|  | pN1 | 3 | 3 | 0 | 0.376 | (≧pN1) |
|  | pN0 | 8 | 4 | 4 | none |  |
| **Stage** | |  |  |  |  |  |
|  | IV | 18 | 11 | 7 | 0.679 | (≧IV) |
|  | III | 6 | 5 | 1 | 0.153 | (≧III) |
|  | II | 4 | 1 | 3 | none | (≧II) |
|  | I | 0 | 0 | 0 | none |  |
| **Sex** | |  |  |  |  |  |
|  | male | 26 | 16 | 10 | 0.64 |  |
|  | female | 2 | 1 | 1 |  |  |
| **Recurrence** | |  |  |  |  |  |
|  | plus | 11 | 10 | 1 | ^a^0.011 |  |
|  | minus | 17 | 7 | 10 |  |  |
| **Age** | |  |  |  |  |  |
|  | average | 67.4 | 68.5 | 65.8 | 0.197 |  |
|  | median | 68 | 68 | 68 |  |  |

^a^Statistically significant.
