# Supplementary material for: CD271 Defines a Stem Cell-Like Population in Hypopharyngeal Cancer
Source: PLoS One. 2013 Apr 23;8(4):e62002. doi: 10.1371/journal.pone.0062002 (PMC3633921; doi:10.1371/journal.pone.0062002)
Supplement: Table S5 — Tumorigenicity of CD44+ and CD44− cells in vivo (HPCM1). In vivo tumorigenesis assay is performed as described in Materials and Methods S1. (DOCX) [file pone.0062002.s011.docx]

**Table S5**. Tumorigenicity of CD44^+^ and CD44^-^ cells in vivo (HPCM1).

|  | cell count | | | | | |
| --- | --- | --- | --- | --- | --- | --- |
| population | | 100 | 300 | 1000 | 3000 | 10000 |
| CD44^+^ | | 3/7 | 5/6 | 3/3 | 1/1 | 1/1 |
| CD44^-^ | | 4/7 | 4/6 | 1/1 | 1/1 | 1/1 |
