# Supplementary material for: CD271 Defines a Stem Cell-Like Population in Hypopharyngeal Cancer
Source: PLoS One. 2013 Apr 23;8(4):e62002. doi: 10.1371/journal.pone.0062002 (PMC3633921; doi:10.1371/journal.pone.0062002)
Supplement: Materials and Methods S1. — (DOCX) [file pone.0062002.s012.docx]

**Supporting Information, Materials and Methods**

***IHC***

Paraffin-embedded, formalin-fixed, 3-μm tissue sections were deparaffinized in xylene, and rehydrated through ethanol to distilled water. Heat-induced epitope retrieval was performed by microwaving sections in a pH 9.0 target retrieval solution (Dako) for MMP10, CK5/6, and CK8, or in a pH 6.0 citrate buffer solution (Mitsubishi Chemical Medicine) for MMP1, MMP2, and Nanog. The endogenous peroxidase was blocked with 0.3% H_2_O_2_. The sections were incubated with primary antibodies for human MMP1 (1:50, Abcam), human MMP2 (1:1000, Abcam), human MMP10 (1:3000, Abcam), human CK5/6 (1:500, Dako), human CK8 (1:100, Dako), or human Nanog (1:800, CST) for 30 min (MMP1, MMP2, MMP10, CK5/6, and CK8) at 37℃, or overnight (Nanog) at 4℃. Mouse LINKER (Dako), or rabbit LINKER (Dako) was applied based on the host species of the primary antibody, then secondary antibodies and DAB Chromogen (Envision^TM^ FLEX Kit, Dako) were applied as described in the manufacturer’s protocol.

***In vivo tumorigenesis assay***

Cells derived from xenotransplanted HPC tumors were sorted based on the human EpCAM and CD44 expression, as EpCAM^+^ CD44^+^ cells or EpCAM^+^ CD44^-^ cells. The sorted cells were then suspended in 200 μl of Matrigel matrix (BD Biosciences) at 4℃, and injected subcutaneously into the flanks of NOG mice with a 1-ml syringe. Each mouse received CD44^+^ cells in the right flank, and CD44^-^ cells in the left. Tumor formation was monitored by weekly inspection and palpation.
